# An educative nutritional intervention supporting older hospital patients to eat sufficiently using eHealth: a mixed methods feasibility and pilot study

**DOI:** 10.1186/s12877-023-04582-x

**Published:** 2024-01-04

**Authors:** Rikke Terp, Lars Kayser, Tove Lindhardt

**Affiliations:** 1 Department of Internal Medicine, Copenhagen University Hospital, Herlev and Gentofte Hospital, Hospitalsvej 1, 2900 Hellerup, Denmark; 2 Department of Public Health, University of Copenhagen, Øster Farimagsgade 5, 1353 Copenhagen K, Denmark

**Keywords:** Feasibility and pilot study, Educative nutritional intervention, eHealth, Malnutrition, Older patients, Patient participation, Self-management

## Abstract

**Background:**

Insufficient food intake is common in older hospital patients and increases the risk of readmission, mortality, and decline in functional status. To improve food intake in older patients, an eHealth solution (Food’n’Go) enabling them to participate in their own nutritional care was implemented in a hospital unit. We developed an educative nutritional intervention (ENI) to support hospitalized older adults (aged ≥ 65 years) to participate in their own nutritional care using Food’n’Go. In this study, we evaluate the feasibility of the ENI and its potential to improve nutritional intake.

**Methods:**

Feasibility was evaluated using process evaluation, and nutritional intake was examined by using a pre- and post-test design. Assessment of *feasibility*: *Contextual factors* (availability of Food’n’Go and prevalence of counseling by a dietitian); *Intervention fidelity* (whether patients were informed of nutrition and Food’n’Go, and whether their needs for support were assessed); and *Mechanism of impact* (patients’ knowledge and skills related to nutrition and the use of Food’n’Go and their acceptance of the ENI). Assessment of *nutritional intake*: Patients’ intake of protein and energy based on one-day observations before implementation of the ENI (pre-test; *n* = 65) and after a three-month intervention (post-test; *n* = 65).

**Results:**

*Feasibility*: Food’n’Go was available for more patients after the intervention (85 vs. 64%, *p* = .004). Most patients managed the use of Food’n’Go and were involved in ordering their food, but only a few monitored their food intake. Information on nutrition was not provided sufficiently to all patients. In general, the ENI had high acceptability among the patients. *Nutritional intake*: Compared to patients in the pre-test, patients in the post-test had a higher daily mean intake of energy (kJ) (6712 (SD: 2964) vs. 5660 (SD: 2432); difference 1052 (95% CI 111–1993)), and of protein (g) (60 (SD: 28) vs. 43 (SD: 19); difference 17 (95% CI 9–26)). Likewise, there was an increase in the mean attainment of protein requirements: 73% (SD: 34) vs. 59% (SD: 29) (*p* = .013).

**Conclusion:**

The ENI is feasible for supporting hospitalized older adults to participate in their own nutrition using eHealth and preliminary results indicate that it may lead to an increasing energy and protein intake.

**Supplementary Information:**

The online version contains supplementary material available at 10.1186/s12877-023-04582-x.

## Background

Sufficient food intake is crucial for hospitalized older adults to maintain or improve their nutritional status, as up to 70% are already at risk of malnutrition at admission [1, 2]. Nevertheless, insufficient food intake and deterioration in nutritional status are commonly described among hospital patients [3–5] and adversely impact patient outcomes, such as physical function [6], risk of readmission [7, 8] and mortality [6, 7, 9–12]. Multiple factors may cause older patients to not eat sufficiently, including a loss of appetite and insufficient awareness and knowledge of the importance of adequate nutrition [13, 14]. Various interventions are recommended to improve their food intake [13, 15]. However, the efficiency of these interventions ultimately depends on individuals' eating behavior, highlighting the importance of including educational activities aimed at behavioral change into nutritional interventions. Digitalization and the growth of new health technologies may be utilized to help individuals actively participate in managing their own health [16], and facilitate behavioral change which is associated with improved health outcomes [17, 18]. However, research on the use of health technology to increase patient participation in own nutritional care among older persons is limited, and it is further constrained in the context of older patients in a hospital setting [19, 20]. 

In﻿ cooperation with older patients and a company (Movesca), our research group developed an app-based eHealth solution called Food’n’Go, designed to support older patients in participating in the effort to eat sufficiently while they are hospitalized [21]. Food’n’Go was made available in a hospital unit in 2017, but the implementation of patient participation was deficient. Audits conducted in 2018 showed that more than 75% of admitted patients were not introduced to Food’n’Go (*Personal communication Terp R. 2023).* Successful adoption and use of an eHealth solution such as Food’n’Go requires that it to be introduced and accompanied by support tailored to the end users’ competence and needs [16, 22]. Therefore, we in a former study developed an educative nutritional intervention (ENI) designed to support older inpatients’ participation in their own nutritional care, assisted by Food’n’Go [23]. Positive results from educative interventions aimed at preventing malnutrition in older, community-living persons have been described in a systematic review by Rea et al. [24], although they concluded that the findings were inconsistent across the included studies. In recommendation on management on nutrition in older persons, education is recommended, however, primarily focusing on information and nutritional counseling [13, 15].

The ENI builds on the findings from previous studies in which we explored barriers and facilitators for involvement of older patients in their own nutritional care by the use of information and communication technology (ICT) [23, 25, 26]. Prevailing barriers among the patients were low self-efficacy regarding use of ICT and lack of nutritional knowledge which were addressed in the ENI. Barriers related to the nursing staff, such as a lack of knowledge, skills, and routines in patient involvement using eHealth, as well as an attitude toward older patients as incapable of using and benefiting from eHealth, were addressed in a plan for educating and supporting the nursing staff. This plan aimed to enable them to conduct the ENI [23].

The effectiveness of the ENI depends on whether it is feasible in daily practice and whether it is perceived as acceptable by patients and healthcare professionals. Furthermore, understanding the context, implementation, and mediating factors is important for the interpretation of the intervention’s outcomes [27, 28]. Here, we aim to evaluate the feasibility of the ENI and its potential to improve nutritional intake among older patients in a hospital unit of internal medicine.

## Materials and methods

### Design

We evaluated feasibility using process evaluation [27, 28] and patients’ food intake using a pre- and post-test design [29]. Participants were informed about the study, and informed consent was obtained when necessary. We used the Template for Intervention Description and Replication (TIDieR) checklist to ensure the transparency and completeness of the reporting [30] (TIDieR checklist provided as Additional file 1).

### Participants, recruitment, and setting

**Fig. 1:**
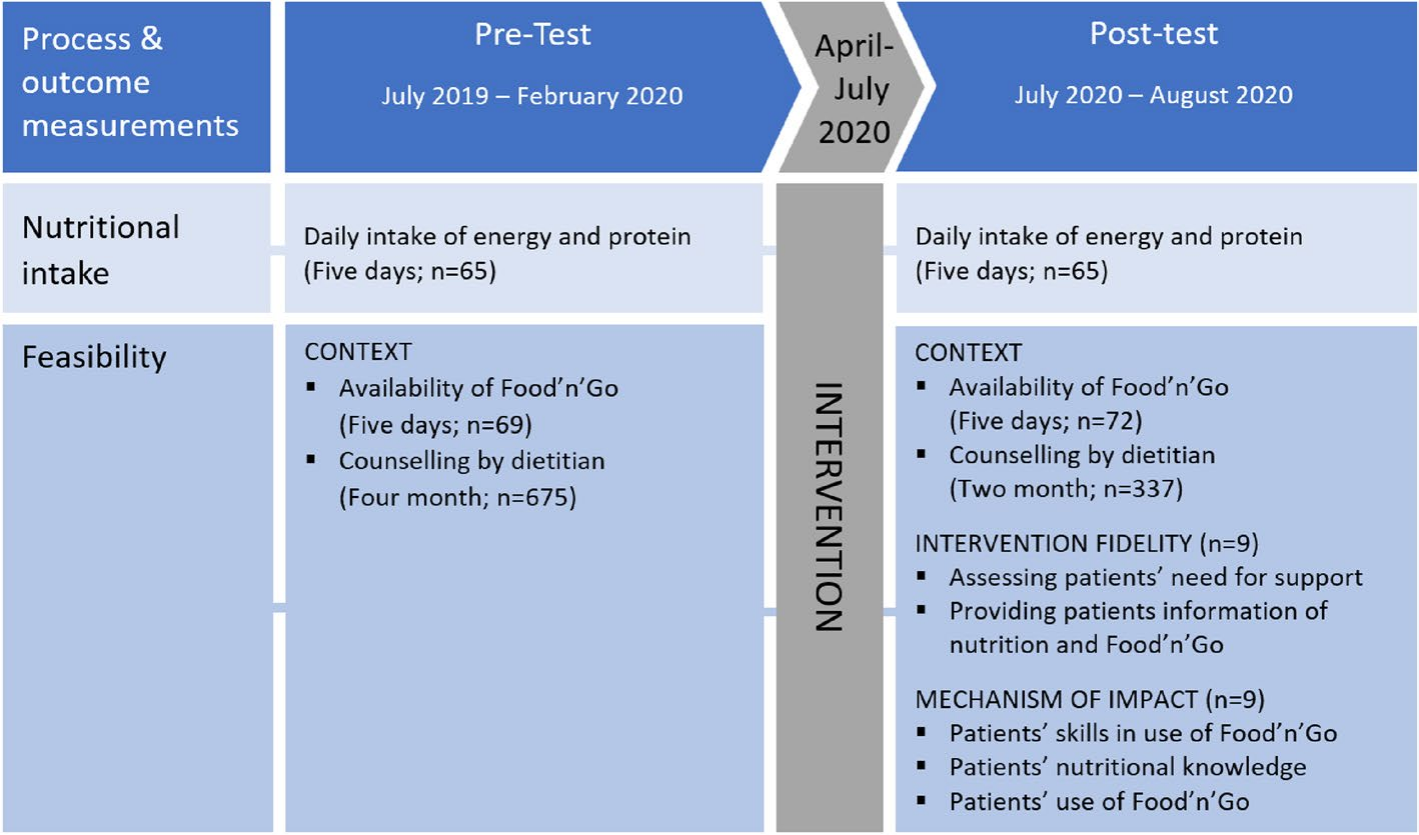
Overview and timeline of the assessments and data collection

This study was conducted in one hospital unit (21 beds) in the Department of Internal Medicine at a university hospital in the capital region of Denmark. Screening and inclusion of patients for the evaluation of feasibility and nutritional intake (post-test), were carried out after a three-month intervention period (April to July 2020). The first author (RT) conducted the screening and inclusion of patients. Timeline for the assessment of feasibility and nutritional intake is illustrated in Fig. 1.

#### Participants included for evaluation of nutritional intake

**Fig. 2:**
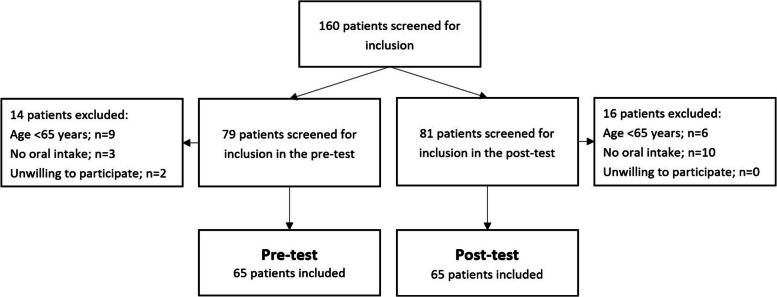
Flowchart for inclusion and exclusion of patients for evaluation of nutritional intake

We included participants using a cross-sectional sampling strategy. On five selected days at both the pre- and the post-test respectively, we screened and included admitted patients at the given time for a one-day observation of their food intake. Patients aged ≥ 65 years who were admitted in time for the breakfast on the observation day were eligible for enrollment. Patients with no oral food intake and who had declined participation in the hospital’s quality improvement activities in general were excluded. Calculation of the sample size was based on the mean attainment of daily estimated energy (kJ) requirements (EER). Based on estimates from former observations of patients’ food intake in the participating unit, we estimated that patients would have a daily mean intake of 45 ± 30% (mean ± SD) of the EER prior to the intervention. After the intervention, we expected patients to reach 60% of their EER. With z-alpha and z-beta (type 1 and type 2 errors) of 5% and 20%, respectively, a sample size of 126—63 in each group— was required. In total, 130 patients were included: 65 patients each in the pre-test and post-test. Figure 2 shows the flow of inclusion and exclusion of patients. No data containing personally identifiable information were collected in the sample for evaluation of nutritional intake, therefor overlap of participants may have occurred between the two groups. We have, however, treated the groups as independent in the statistical analysis.

#### Participants included for feasibility evaluation (Intervention fidelity and mechanism of impact)

**Fig. 3:**
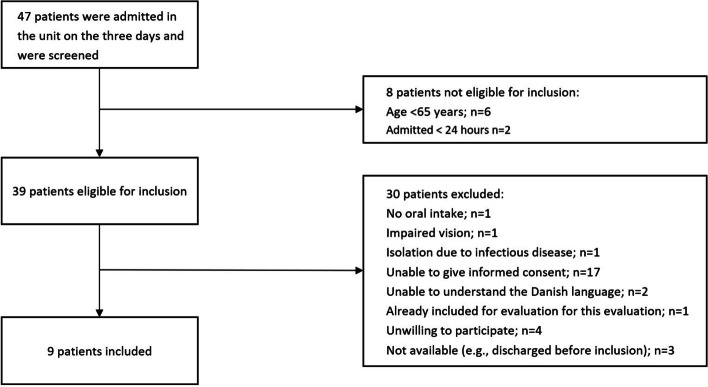
Flowchart for inclusion and exclusion of patients for evaluation of fidelity and mechanism of impact

Screening, inclusion and data collection on fidelity, and the mechanism of impact were conducted on the days following a one-day observation of patients' food intake (post-test), in total three days. On the three days, all patients admitted to the unit were screened for eligibility. Patients aged ≥ 65 years and hospitalized in the unit for more than one day were eligible for enrollment. Exclusion criteria were no oral food intake, isolation due to infectious disease, severe cognitive impairment impeding provision of informed consent, lack of understanding of the Danish language, or inability to use Food’n’Go due to impaired vision. We screened 39 patients for eligibility and excluded 30; in total, nine patients, three of whom were women, were included (Fig. 3).

### Intervention

#### ENI

**Fig. 4:**
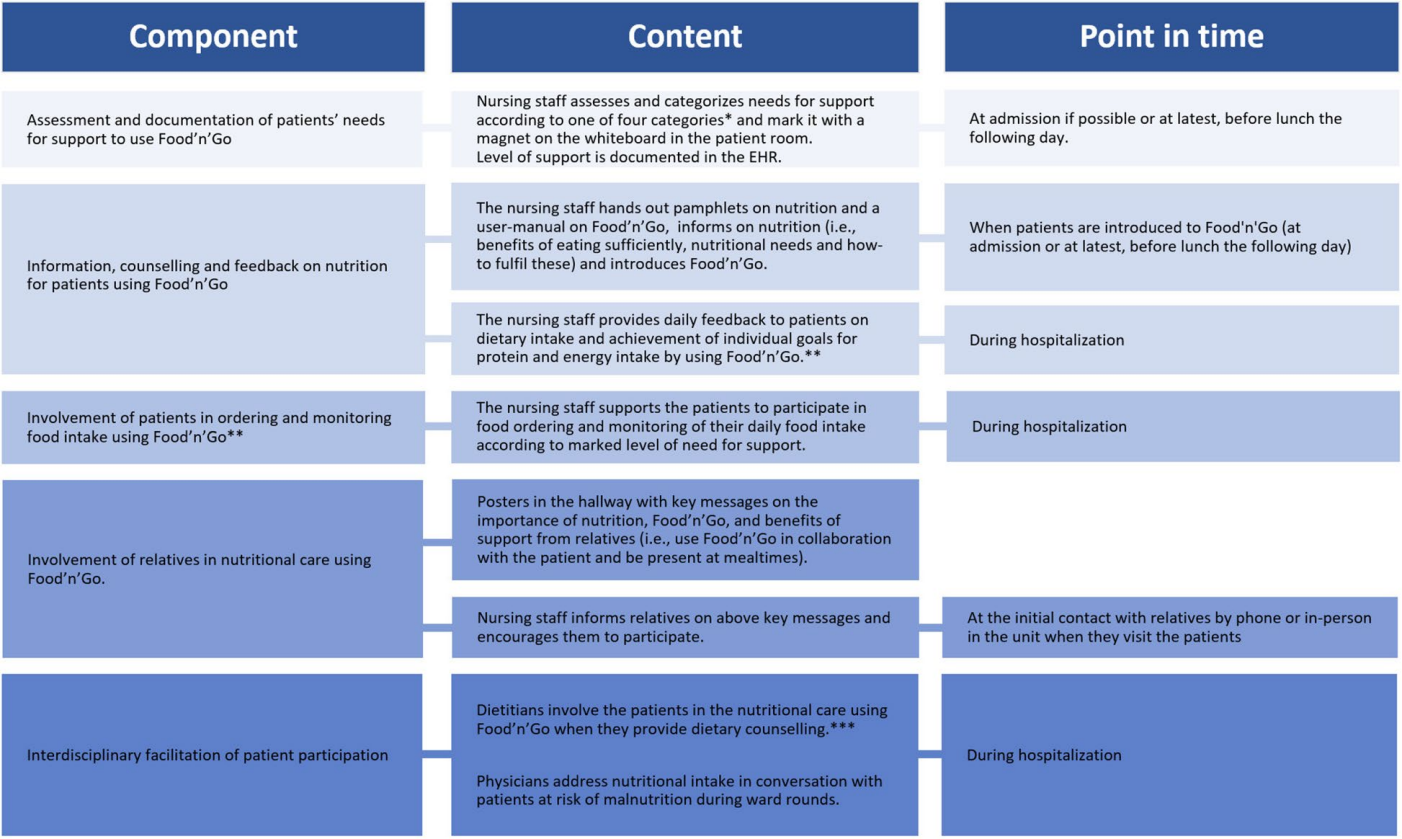
The Educative Nutritional Intervention—ENI* (1) Patient is able to hold and operate the tablet with Food’n’Go without support; (2) Patient is able to hold and operate the tablet with Food’n’Go with verbal and/or technical support; (3) Patient is able to participate in use of Food’n’Go when the tablet is held and operated by another; (4) Patient is not able to participate in use of Food’n’Go. ** Monitoring of food intake was only required for patients at risk of malnutrition (NRS ≥ 3). *** Dietary counselling by dietitian for patients at risk of malnutrition was a part of standard nutritional care and was in this ENI extended to include use of Food’n’Go. *Source:* Terp et al. Theory-driven development of an educative nutritional intervention (ENI) supporting older hospital patients to eat sufficiently, assisted by an eHealth solution: an intervention mapping approach. BMC Health Serv Res. 2022 Dec 1;22(1):1–15

The ENI consists of five components describing how healthcare professionals and relatives can support older patients to participate in their own nutritional care using Food’n’Go during hospitalization. An overview of the ENI is shown in Fig. 4. The ENI was developed in cooperation with older patients (aged ≥ 65 years), relatives, and healthcare professionals. It is based on a previous study exploring older patients’ competence, needs, and attitudes toward nutrition and the use of ICT [25, 26]; recommendations for nutritional management in older patients [13, 15]; theories of behavioral change [31–34]; and readiness to engage with health technology [35, 36]. The development process of the ENI was reported in a previous article [23]. During a three-month intervention period, the ENI was offered to all patients in addition to the standard nutritional care described below, and several educational activities targeting healthcare professionals took place to enhance their knowledge, skills, and attitudes regarding delivery of the ENI. A detailed description of these educational activities is described in a previous article [23]. Due to the Covid-19 pandemic, there was, at times, no or restricted access to the unit for relatives, which is why the component of ENI involving relatives was not implemented or evaluated. A template for nursing documentation in the electronic health record (EHR) was developed; however, it was not released for use in the EHR system until after the three-month period, and thus neither implemented nor evaluated in this study.

#### Food’n’Go technology

The Food’n’Go solution is an android-based app connected to a Microsoft backbone system and installed on a Lenovo tablet with a 10.1" screen (TB2-X30L) with a 4G telco connection. The data were hosted in a Microsoft-based backbone system, which is hosted in a secure environment behind a firewall. All connections between the tablet and the Microsoft backbone system were encrypted with a secure sockets layer (SLL), with an SHA256 + RSA signature algorithm, using a 2048-bit key. Food’n’Go contains functions by allowing the patients to 1) view photos of the hospital’s selection of foods and drinks, 2) select and order foods and drinks, 3) receive feedback about whether their order is satisfying according to their protein requirements, 4) record intake of food and drinks, and 5) receive feedback on their daily energy and protein intake. At the back end, the nurse or dietitian enters the patient’s estimated energy requirements (EER) and estimated protein requirements (EPR), which enables the patient to view the attainment of their requirements of the day on a bar chart on the tablet. Food’n’Go was implemented in the participating unit in 2017 and made available to all patients on a tablet placed in the patient room. In 2017, food ordering and monitoring of food intake was carried out through Food’n’Go, regardless of whether it was managed by the patients or the nursing staff. From 2018 to the end of 2019, a project nurse was employed to facilitate the implementation of Food’n’Go.

#### Standard nutritional care

The standard procedure for the management of nutrition provides the following instructions to the nursing staff: Screening for malnutrition within 24 h after admission using the Nutritional Risk Screening (NRS-2002) [37]. Patients were identified as at risk of malnutrition by an NRS score ≥ 3, and standard recommendations for nutritional care for patients at risk were assessment of EER and EPR, daily monitoring of their dietary intake (energy and protein), adjustment of the plan for dietary intake if necessary, and referral to a dietitian for further assessment and counseling if the patient was unable to reach their required energy and protein needs EER and EPR.

### Assessment and procedure

The timeline of the collection on data for evaluation of feasibility and nutritional intake is illustrated in Fig. 1. In the pre-test, data was collected prior to the implementation of the ENI, and in the post-test after the three-month intervention period.

#### Assessment of feasibility

The ENI consists of several interrelated components and is thus a complex intervention [27]. Inspired by the framework for process evaluation recommended by the Medical Research Council (MRC) for complex interventions [27, 28], we explored the feasibility according to the following three themes: C*ontext*; factors external to the intervention that affects the implementation or intervention outcome; *Fidelity* of the intervention; and *Mechanism of impact* on intervention outcomes. 

##### Context

The availability of Food’n’Go for patients with oral intake and the proportion of patients receiving dietary counseling from a dietitian during hospitalization were assessed before implementation of the ENI and after the three-month intervention period. Availability of Food’n’Go was defined as a charged tablet at the patients’ disposal, and the patient in question being logged into the system. It was assessed by reviewing all patients and recording whether Food’n’Go was available. The first author RT, assisted by the project nurse, conducted these observations. Data on the proportion of patients receiving dietary counseling by a dietitian were recorded from the EHR; however, these data did not reveal whether the counseling had involved using Food’n’Go as prescribed in the ENI.

##### Intervention fidelity

The delivery of the ENI was registered according to I) whether the patients’ needs for support were marked with one of four magnets on their whiteboard, each representing four different levels of support for using Food’n’Go (see Fig. 4); II) agreement between the nursing staff’s and the observers’ assessment of the level of need for support; III) whether the patients were provided with verbal and written information on nutrition; and IV) whether they were introduced to Food’n’Go. I–IV were assessed using observation and structured individual interviews with patients conducted by the first author.

##### Mechanism of impact

The mechanism of impact was assessed according to the patients’ V) *participation* in the use of Food’n’Go; VI) *knowledge* of nutrition; VII) *skills* related to the use of Food’n’Go; and VIII) perception of the *acceptability* of the nutritional intervention, including the use of Food’n’Go. V and VIII were assessed using individual semi-structured interviews. An interview guide inspired by the theoretical framework for acceptability by Sekhorn et al. [38] was developed comprising the acceptability components *affective attitude* (how the patients felt about the intervention) and *perceived effectiveness* (whether the intervention affected their food intake). Their *knowledge* and *skills* (VI and VII) were assessed using an Objective Structured Clinical Examination (OSCE) approach [39] as described below. In addition to the OSCE, an open question was asked addressing the patients’ knowledge of their nutritional needs while they were acutely ill. The observation and interview guide were pilot-tested on three patients, leading to minor changes in the introduction of the nutritional knowledge test.

#### OSCE of the patients’ knowledge and skills (VI and VII)

Each patient was presented with two posters containing nine pictures of food and drink items with varying content of energy and protein (low, medium, and high), and asked to select the three food or drink items with the highest content. Knowledge was evaluated based on the sum of kilojoules (kJ) and grams (g) of protein in the three selected pictures, calculated as a percentage of the ideal choice. After this, the patients were asked to perform two tasks that assessed their skills in using Food’n’Go: ordering two items of food or drink and registering these items as consumed. The observer (first author) registered the extent to which they could complete the tasks without support, and field notes about patients’ comments and reactions were taken.

#### Assessment of nutritional intake

The patients’ nutritional intake was based on one-day observations and measured as 1) daily mean intake of energy (kJ) and protein (g) and 2) percentage achieved of daily EER and EPR, with ≥ 75% as the goal [40]. The intake was observed by the first author with assistance from a project nurse. We registered all food and drinks that were served from 7:00 AM to 9:00 PM and the amount consumed, measured as 100, 75, 50, 25, or 0%. The intake from the three main courses was assessed using a print of the food order and a photo of the food tray after the meal. The first author adjusted the food order print according to what was served in case of deviation from the original order due to the patient’s wishes. To ensure reliability of the assessment of the food intake, the consumption of 10 served meals (five from pre-test and five from post-test) was evaluated by a dietitian blinded to group affiliation. The test showed 100% agreement in 8 of 10 meals. In two meals, minor disagreements resulting in a difference of 33 and 188 kJ were discussed, and consensus was achieved in favor of the assessment conducted by the first author. Data on the patients’ EER and EPR were drawn from the EHR, in which the estimates were calculated based on body weight, physical activity, and temperature [41]. The intake was distributed over three main meals (23%, 27%, 24%) and three in-between meals (13%, 9%, 4%). The intake for patients who were discharged during the day of our observation was estimated as a percentage corresponding to a full day’s intake.

### Data analysis

#### Qualitative analysis

Data from structured interviews (I, III, IV, V) were categorized and semi-structured interviews (VIII) were analyzed using deductive content analysis [42, 43]. Data regarding acceptability were coded and analyzed according to the two acceptability components *affective attitude* and *perceived effectiveness* [38]. The analysis was conducted by the first and last authors and discussed with the second author afterwards.

#### Statistical analysis

Descriptive and analytical statistics were applied using SPSS version 25 (Armonk, NY: IBM Corp.). Nutritional intake of energy and protein was measured as a continuous variable for total mean intake per day and a dichotomized variable of a minimum intake of 75% of requirements per day. Comparison of means of continuous variables using the students t-test, and for categorical variables using the chi-square tests. A *p*-value of less than 0.05 was considered statistically significant.

## Results

### Feasibility

#### Context: Availability of Food’n’Go and dietary counseling by a dietitian

A significant increase in availability of the tablet with Food’n’Go was found after the intervention. In the post-test, Food’n’Go was available for 61 out of 72 (85%) patients, compared to 44 out of 69 (64%) in the pre-test (*p* = 0.004). More patients received dietary counseling from a dietitian after the intervention (41 out of 337 (12%)) than before the intervention (54 out of 675 (8%)) (*p* = 0.032).

#### Intervention fidelity and mechanism of impact

The included patients’ ages ranged from 73 to 94, the mean length of stay in the unit when interviewed was 4.4 days, and five of the nine patients were at risk of malnutrition (NRS ≥ 3). None of the nine included patients had received dietary counseling by a dietitian during their hospitalization. In Table 1, the data on fidelity and mechanism of impact are summarized. Based on the nursing staffs’ assessment, the patients’ competence and need for support to use Food’n’Go ranged from “able to use Food’n’Go with no support” (four patients), “able to use Food’n’Go and operate the tablet with support” (one patient) and “able to participate in use of Food’n’Go when the tablet is held and operated by the nursing staff” (three patients). All except one patient reported using Food’n’Go to order food, while only three used it to monitor their intake. Four patients reported that the nursing staff operated the tablet for them. However, the test (OSCE) of the informants’ *skills* in using Food’n’Go showed that all except one was able to operate the tablet, either by themselves or with minor support. *Knowledge* scores on food regarding the content of energy were higher than those on protein. Field notes revealed that some patients did not know what protein was. The interviews revealed that all patients knew that food was important for their health, but misconceptions appeared regarding how to fulfill their nutritional needs. For instance, some emphasized vegetables and taste, while only a few understood the importance of protein (Patient D, E, and G).

**Table 1:** Intervention fidelity and mechanism of impact

Variables	Participants (*N* = 9)								
	A	B	C	D	E	F	G	H	I
**Intervention fidelity**									
Patient category – level of need for support^a^									
Assessed by nursing staff	1	1	1	3	1	3	2	-^b^	3
Assessed by first author	1	1	1	1	1	1	2	1	2
Received information from the nursing staff									
Pamphlet of nutrition	Yes	Yes	Yes	Yes	Yes	No	No	No	No
Information of nutrition	No	Yes	No	No	Yes	No	Yes	No	No
Introduced to Food’n’Go	Yes	Yes	Yes	Yes	Yes	Yes	Yes	No	Yes
**Mechanism of impact**									
Use of Food’n’Go during hospitalization									
Participate in food ordering									
*Yes – operate the tablet*	X	X	X	X	X				
*Yes – nursing staff operate the table*						X	X		X
*No*								X	
Participate in monitoring of food intake^c^									
*Yes – operate the tablet*	X		X	X					
*Yes – nursing staff operate the table*									
*No*		X			X	X	X	X	X
Nutritional knowledge score in percentage^d^									
Knowledge of calorie content	100	100	100	86	89	86	100	63	78
Knowledge of protein content	100	85	37	74	100	80	74	38	100
Skills in use of Food’n’Go^e^									
Food ordering	1	1	1	1	1	1	2	1	1
Monitoring food intake	1	1	1	1	3	1	2	1	2

^a^1 = Patient is able to use Food’n’Go without support, 2 = Patient is able to use Food’n’Go and operate the tablet with support, 3 = Patient is able to participate in use of Food’n’Go when the tablet is held and operated by the nursing staff; 4 = Patient is not able to participate in use of Food’n’Go

^b^No magnet on the patient’s white board

^c^Monitoring of food intake was only required in patients with a NRS score ≥ 3, which was identified for informant B, D,G; H and I

^d^100% is the best achievable score

^e^1 = Yes – operate the tablet without support; 2 = Yes – operate the tablet with minor support; 3 = Yes – operate the tablet with major support; 4 = No

The qualitative analysis of *acceptability* in terms of the component *affective attitude* showed that the patients appreciated being involved in their own nutritional care. A prevailing finding was the patients’ positive experience with individualized support from nursing staff, such as guidance on food intake and encouragement to eat despite a lack of appetite. They valued the focus on their food preferences when encouraged to eat sufficiently. Some patients appreciated the information on nutrition and would have liked to be more informed, while others were not interested or felt no need for such information.

You read about what you need to know. (Patient A)

The patients were satisfied with Food’n’Go as a tool for participation in their nutritional care, as it provided them with the opportunity to order the food they liked. Different kinds of support regarding the use of Food’n’Go were mentioned. The patients’ narratives revealed variations in how much the nursing staff expected and encouraged them to participate in the use of Food’n’Go, and this was apparently accepted by the patients. One patient had learned to use the system well by himself and had, despite extensive technical challenges (e.g., unstable internet access), used Food’n’Go during his hospital stay. He was annoyed that he had not received the user guide for Food’n’Go, as it would have made it easier for him. Furthermore, he reported a tendency among the staff to take over when technical problems appeared instead of teaching him to solve them himself.

They are quick. If I don’t figure it out immediately, they fix it quickly. They don’t just show me. (Patient B)

In terms of the acceptability component *perceived effectiveness,* the impact of the food’s presentation and taste on the patients’ motivation to eat was dominant in all interviews. When asked about how the use of Food’n’Go influenced their food intake, several emphasized how inviting photos of the food enhanced their motivation to order and eat despite a lack of appetite. In general, the possibility of participating in monitoring their food intake was not mentioned. Some expressed indifference to the feedback diagrams on their intake in Food’n’Go when asked directly. However, one patient expressed that it was “smart” with a chart providing feedback on the food intake, as it prevented them from eating too much.

### Nutritional intake

Table 2 shows the patient characteristics and the served and consumed energy and protein. No differences in patient characteristics were found except daily EPR, which was higher among patients in the post-test than in the pre-test. The patients in the post-test were served food with significantly more energy and protein than those in the pre-test. The intake was also improved for both energy and protein. Furthermore, the attainment of the patients’ EER and EPR increased from the pre-test to the post-test, significantly so for protein. Although less than half of the sample in the post-test reached the goal of 75% intake for protein, significantly more reached the goal than in the pre-test. A higher percentage reached the goal for energy (kJ), although no significant difference between the pre- and post-test was established.

**Table 2:** Characteristics, nutritional status, and nutritional intake of patients before and after the intervention

	**Pre-test (*N* = 65)**	**Post-test (*N* = 65)**	***P*-value**^**a**^	**Difference (mean) (CI 95%)**
**Age (years), mean (SD)**^b^	82 (7.2)	83 (5.8)	.421	
**Sex (female), n (%)**	30 (44)	39 (57)	.114	
**Discharged during the day**	17 (26)	10 (15)	.113	
**Nutritional status**				
BMI^c^, mean (SD)	24.1 (6.04)	25.2 (5.2)	.277	
EER^d^ (kJ/day), mean (SD)	8136 (1455)	8542 (1531)	.122	
EPR^e^ (g/day), mean (SD)	85 (17)	91 (16)	.049	
**Served food and drinks**				
Total energy (kJ)/day, mean (SD)	9009 (2890)	10858 (3512)	.001	1849 (732–2965)
Total Protein (g)/day, mean (SD)	68 (23)	95 (35)	.000	26 (16–37)
**Nutritional intake**				
Total energy (kJ)/day, mean (SD)	5660 (2432)	6712 (2964)	.029	1052 (111–1993)
Total protein (g)/day, mean (SD)	43 (19)	60 (28)	.000	17 (9–26)
Attainment of EER, % mean (SD)	79 (37)	88 (42)	.212	9 (-8–22)
Attainment of EPR, % mean (SD)	59 (29)	73 (34)	.013	14 (3–25)
Intake > 75% of EER, N (%)	36 (55)	39 (60)	.594	
Intake > 75% of EPR, N (%)	17 (26)	29 (45)	.028	

^a^Pearson’s chi-square test was used for categorical variables and students’ t-test for continuous variables

^b^*SD* Standard deviation

^c^*BMI* Body mass index

^d^*EER* Estimated energy requirements

^e^*EPR* Estimated protein requirements

## Discussion

### Feasibility

Overall, the ENI was evaluated as feasible and considered acceptable by the patients. Limited evidence exists on the involvement of older patients in their own care during hospitalization through the use of health technology [44]. Consistent with other studies [21, 45], the patients were satisfied with using the eHealth solution Food’n’Go as a tool for participation in their own nutritional care. The ENI is a multicomponent intervention aiming to address personal determinants for management of nutrition and the use of eHealth; that is, ensuring patients have adequate knowledge and skills and feel confident in engaging with Food’n’Go. While almost all patients reported that they had been introduced to Food’n’Go, only a few reported having received information on nutrition. Despite this, the participating patients perceived food intake to be important, and had sufficient knowledge to identify food and drinks with high energy content. However, they were not aware of the benefits of eating energy- and protein-rich foods, and they had challenges identifying foods with high protein content. Notably, the informants who emphasized the need for healthy food with vegetables also answered that they had not received information on nutrition. This indicates that an increased focus on providing patients with knowledge of their dietary needs for protein and how to fulfill these is important.

The evaluation showed that the patients perceived their participation in their nutritional care using Food’n’Go as acceptable. However, there were some challenges that need to be addressed in the future use of ENI. For instance, the results on fidelity indicated inadequate involvement of the patients in monitoring their food intake, which prevented them from getting feedback and the intended motivational effect. This may explain the patients’ complacency toward the feedback function with the diagram of their nutritional intake. Feedback and participation in monitoring were expected to have an impact on the patients’ motivation to eat sufficiently, based on the behavioral change theory of goal-setting and self-monitoring as techniques to promote behavior change [34]. Findings from our previous study showed that older persons were not experienced with monitoring their nutritional status or their food intake, and, in general, they considered food intake an everyday phenomenon valued as a social and sensory activity [25]. This emphasizes the need for support with a focus on the monitoring part if patient participation is to be achieved.

Several studies have reported a lack of involvement of older hospitalized patients [46, 47], and barriers are often related to healthcare professionals, including their perception of time constraints, preconception of who is to benefit from active participation, and lack of skills for facilitating patient participation [46–48]. This underlines that a prerequisite for successful patient participation is to ensure that healthcare professionals have adequate knowledge and skills to engage with patients in full partnership. Purchasing and introducing eHealth aimed at enhancing patient participation without ensuring the necessary competencies among healthcare professionals can be economically costly and inefficient. To ensure successful and sustainable implementation of eHealth, organizations need to allocate human resources for training and supporting healthcare professionals, enabling them to involve and support the patients. In terms of the economic aspect, we argue that the ENI and the educational activities targeting nursing staff in this study are cost-effective in the long term.

According to the Technology Acceptance Model 3 (TAM3), an individual’s perception of a technology’s ease of use and usefulness are important determinants of IT adoption and use [36]. These determinants are addressed in the ENI by providing information on the benefits of eating sufficiently, enhancing social support from healthcare professionals and relatives, and involving and supporting patients in their use of Food’n’Go according to their competence and needs. An essential part of the ENI was individualized involvement and support according to the patient’s competence and needs. The nursing staff were instructed to assess patients’ levels of need for support for using Food’n’Go according to four defined levels and mark it with a magnet on the patients’ whiteboard. Cases with disagreement regarding the level of need for support when assessed by the first author indicated that the nursing staff assessed a higher need for support than was necessary. Nurses’ assessment skills and need for training should be monitored in the future use of the ENI.

Examinations of the patients’ skills in using Food’n’Go showed that all could operate the tablet themselves, and the majority without any support. In a former study, observations conducted in the hospital unit showed that 50% of patients were able to use Food'n'Go when the tablet was held and maneuvered by the nursing staff [23]. It is noteworthy that the sample may not be representative of the general population of older hospitalized patients, as willingness and ability to participate in the study probably excluded the most frail patients. In future research, it is important to acquire further knowledge about the group of older patients who require the most support to actively participate in their own nutrition using health technology, as this is likely the group at the highest risk of malnutrition. We used a cross-sectional sampling strategy. For future research, it should be considered to utilize a purposive sampling strategy to ensure heterogeneity in terms of the patients' technology readiness and competence when evaluating feasibility.

Availability of the tablet with Food’n’Go by the patient’s bed increased markedly in the post-test. Availability of ready and functioning technology is the first precondition for patients to participate in their nutritional care using eHealth, and the improvement reflects a recognition of this among the staff. However, access to Food’n’Go on a non-removable screen in the patient rooms would have been preferable and may be a way to enhance the fidelity of the intervention.

The ENI is intended to create an environment involving formal and informal caregivers to support older patients to be aware of the importance of eating sufficiently and to be motivated to participate using Food’n’Go. According to the intervention fidelity, it is important to note that, due to the Covid-19 situation, relatives were not involved as prescribed in the ENI, which is unfortunate since social support is described as an important determinant for readiness for engaging with eHealth [22, 35, 49]. Hence, the feasibility of involving relatives in nutritional care in a hospital setting needs to be explored in future research.

### Nutritional intake

Patients admitted after implementation of the ENI (the post-test) had a higher intake of energy and protein than patients admitted before (the pre-test). Despite a significantly higher EPR, more patients in the post-test reached the goal of ≥ 75% of their EPR, which indicates the potential of the ENI to improve nutritional intake. In other intervention studies, comparable results on nutritional intake among medical hospital patients have been reported [50, 51]. In a Swiss multicenter study randomized controlled trial (*N* = 2088), Schuetz et al. [51] found a daily energy intake of 1501 kcal (equivalent to 6288 kJ) and protein intake of 47 g among medical hospital patients receiving individualized nutritional support. Although the daily intake of energy and protein were higher among patients in our post-test than reported by Schuetz et al. [51], the attainment of EER and EPR were smaller in our study: 60% reached > 75% of EER and 45% reached > 75% of EPR vs. 79% of EER and 76% of EPR. It is noteworthy that the population in the study by Schuetz et al. differed, as participants aged < 65 years were also included, and they were all malnourished or at risk of malnutrition.

In another Danish study, Pedersen found a significant increase in energy and protein intake among older hospital patients aged (*N* = 253; mean age 76 years) through active patient involvement in their own nutritional care [52]. Pedersen reported daily energy and protein intakes of 6539 kJ and 68 g, respectively, which is comparable to the intake we found among patients in the post-test (6712 kJ and 60 g protein). There are several similarities between the ENI and the intervention in the study of Pedersen regarding the use of behavioral change methods (e.g., providing knowledge, individualized feedback, and self-monitoring) to enhance patients’ motivation [52]. However, in our study, the patients’ participation was assisted by eHealth.

More than one-third of the patients in our study did not achieve their dietary goals for energy intake despite their relatively large intake, and this may be explained by the method used for calculating the EERs. The calculation was performed automatically in the EHR system based on body weight, physical activity, and temperature and not corrected for gender and age, as in the Harris-Benedict equation [53]. Consequently, the EER in our study may have been over-estimated compared to other studies using the Harris-Benedict equation, such as in the study by Schuetz et al. [51].

A major strength of this study was the use of a theory- and evidence-based intervention (ENI). Furthermore, the multiple methods design using quantitative and qualitative methods is a strength and is recommended for the evaluation of complex interventions [27, 28]. The evaluation of feasibility may be beneficial in informing future implementation of the ENI in other settings. The samples included in this study had a relatively high age. This provides us with important knowledge, as the oldest and most frail are often excluded from studies, and consequently, we lack evidence of this patient group, whom we need to involve and support.

This study also has some relevant limitations. First, a randomized controlled trial would have been preferable, but due to methodological issues, we did not choose this design. Second, the evaluation of feasibility (intervention fidelity and mechanism of impact) was based on a small sample with only nine patients but represent all eligible patients available for inclusion on the three days data were planned to be gathered. The reasons for exclusion were mainly due to patients being incapable of providing informed consent due to physical disabilities or cognitive impairment. Furthermore, the majority of the patients included in the feasibility evaluation (for intervention fidelity and mechanism of impact) were able to use Food'n'Go independently or with minimal support. Therefore, our sample may not be representative of the frailest patients, which should be taken into account when considering the transferability of the feasibility evaluation results. Third, we did not address the ENI directly when asking about the patients’ perception of acceptability but sought to gain insight into their experiences and attitudes by addressing nutritional care in general, including Food’n’Go, which we assumed would be easier for patients to talk about and thus provide more meaningful answers. Fourth, this study indicates that ENI may lead to an increase in energy and protein intake. ENI is designed and developed to facilitate behavioral change. However, due to the study design, it is not possible to make any claims about long-term effects. ENI is developed to ensure that patients eat sufficiently during their hospitalization, thereby improving or preventing a decline in their nutritional status during their hospital stay. Since inadequate food intake is often a problem that persists after hospitalization, it is relevant in future research to explore how an educational intervention like ENI can be applied after discharge, in collaboration between the hospital and the primary sector.

## Conclusion

This study shows that the ENI is feasible in clinical practice to enhance older patients’ participation in their own nutritional care by using an existing eHealth solution (Food’n’Go), and preliminary results indicate that it may lead to an increase in energy and protein intake and fulfillment of protein requirements in older hospital patients. This study strengthens the evidence for eHealth as an appropriate method to enhance the participation of older patients in their own nutritional care despite frailty and limited experience with eHealth. In future research, it is important to investigate whether these results may also lead to an improvement in patient-relevant outcomes, such as physical functioning, quality of life, and readmission.

### Supplementary Information﻿

**Additional file 1. **The TIDieR (Template for Intervention Description and Replication) Checklist*

## Data Availability

Data containing personal information cannot be disclosed due to the rules of the General Data Protection Regulation, but author RT can be contacted about any possibilities of seeing aggregated or anonymized parts of the dataset.

## Abbreviations

EER: Estimated energy requirements
EPR: Estimated protein requirements
EHR: Electronic health record
ENI: Educative nutritional intervention
g: Grams
ICT: Information and communication technology
kJ: Kilojoules
MRC: Medical Research Council
NRS: Nutritional risk score
READHY: Readiness and enablement index for health technology
SD: Standard deviation
TAM3: Technology Acceptance Model 3
TIDieR: Template for Intervention Description and Replication
