# Supplementary material for: An educative nutritional intervention supporting older hospital patients to eat sufficiently using eHealth: a mixed methods feasibility and pilot study
Source: BMC Geriatr. 2024 Jan 4;24:22. doi: 10.1186/s12877-023-04582-x (PMC10768306; doi:10.1186/s12877-023-04582-x)
Supplement: Supplementary file 1 — Additional file 1. The TIDieR (Template for Intervention Description and Replication) Checklist* [file 12877_2023_4582_MOESM1_ESM.pdf]

Additional file 1

The TIDieR (Template for Intervention Description and Replication) Checklist*:

Information to include when describing an intervention and the location of the information

Item number	Item	Where located **	
		Primary paper[1] (page or appendix number)	Other [2]
	**BRIEF NAME**		
**1.**	Provide the name or a phrase that describes the intervention.	Educative nutritional intervention (ENI) Referred to in abstract, page 2 onwards	
	**WHY**		
**2.**	Describe any rationale, theory, or goal of the elements essential to the intervention.	Page 4 & 6	Page 2, 6-9, Table 1 & 2, Additional file 2
	**WHAT**		

[1] Primary paper: Terp R, Kayser L, Lindhardt T. An educative nutritional intervention supporting older hospital patients to eat sufficiently using eHealth: A mixed methods feasibility and pilot study. Submitted to BMC Geriatric 2022.

[2] Paper: Terp R, Lindhardt T, Kayser L. Theory-driven development of an educative nutritional intervention (ENI) supporting older hospital patients to eat sufficiently, assisted by an eHealth solution: an intervention mapping approach. BMC Health Service Research. 2022; Accepted for publication 14.10.2022

**3.**	Materials: Describe any physical or informational materials used in the intervention, including those provided to participants or used in intervention delivery or in training of intervention providers. Provide information on where the materials can be accessed (e.g. online appendix, URL).	Page 6, Fig. 1	Page 7-9, Table 2, Additional file 2
**4.**	Procedures: Describe each of the procedures, activities, and/or processes used in the intervention, including any enabling or support activities.	Fig. 1	Page 9-10, Table 2, Additional file 2
	**WHO PROVIDED**		
**5.**	For each category of intervention provider (e.g. psychologist, nursing assistant), describe their expertise, background and any specific training given.		3 & 9
	**HOW**		
**6.**	Describe the modes of delivery (e.g. face-to-face or by some other mechanism, such as internet or telephone) of the intervention and whether it was provided individually or in a group.	Page 6, Fig 1.	Table 2, Additional file 2
	**WHERE**		
**7.**	Describe the type(s) of location(s) where the intervention occurred, including any necessary infrastructure or relevant features.	Page 4-5	Page 2-3
	**WHEN and HOW MUCH**		
**8.**	Describe the number of times the intervention was delivered and over what period of time including the number of sessions, their schedule, and their duration, intensity or dose.	Page 6, 11-12	
	**TAILORING**		
**9.**	If the intervention was planned to be personalised, titrated or adapted, then describe what, why, when, and how.	Fig. 1	Table 1, Additional file 1
	**MODIFICATIONS**		
**10.**[‡]	If the intervention was modified during the course of the study, describe the changes (what, why, when, and how).	6	
	**HOW WELL**		

**11.**	Planned: If intervention adherence or fidelity was assessed, describe how and by whom, and if any strategies were used to maintain or improve fidelity, describe them.	8 (Assessment of fidelity)	Table 2, Additional file 2 (strategies to improve fidelity)
**12.**[‡]	Actual: If intervention adherence or fidelity was assessed, describe the extent to which the intervention was delivered as planned.	Page 11, Table 1	
